# Risk work or resilience work? A qualitative study with community health workers negotiating the tensions between biomedical and community-based forms of health promotion in the United Kingdom

**DOI:** 10.1371/journal.pone.0220109

**Published:** 2019-07-29

**Authors:** Nicola K. Gale, Manbinder S. Sidhu

**Affiliations:** Health Services Management Centre, School of Social Policy, College of Social Sciences, University of Birmingham, Birmingham, United Kingdom; University of Toronto, CANADA

## Abstract

Emplaced health promotion interventions, delivered by community health workers are increasingly being used internationally. However, the application of epidemiological risk knowledge to individuals within such communities is not straightforward and creates tensions for community health workers who are part of the communities that they are serving. Situated qualitative interview data were co-produced with community health workers employed in a superdiverse, deprived, post-industrial region of the United Kingdom, using photo-voice methods, to develop an account of how they made sense of the challenges of their work. The analysis draws on and develops theories of risk work and resilience work, which draw on practice theory. The key findings were that, first, being a *critical insider* enabled community health workers to make sense of the diverse constraints on health and lifestyles within their community. Second, they understood their own public health role as limited by operating within this context, so they articulated their occupational identity as focused on supporting clients to make *small but sustainable* changes to their own and their families’ lifestyles. Third, the uncertainties of translating population based risk information to individual clients were (at least partially) resolved at an embodied level, with the community health workers identifying as accessible and trusted *role models* for the value of changed lifestyles. The article is important for policy and practice as it provides a critique of a rapidly evolving new mode of delivery of public health services, and insights on the development of this new public health workforce.

## Introduction

While the weight of evidence suggests that deprivation and other dimensions of ‘risk’–along the lines of gender, ethnicity, class etc.–consistently produce poor health outcomes, it is notable that some communities have been identified as ‘resilient’; various factors, such as greater availability of social housing or higher concentrations of minority ethnic groups, are seen to have a protective effect on health[1–3]. Given this variation, it seems logical that policy makers might attempt to intervene to reduce specific risks or increase the resilience of particular disadvantaged communities. A growing trend globally has been the use of community (or ‘lay’) health workers (CHWs) to offer emplaced health promotion interventions in the form of ‘health-related lifestyle advice’[4,5] and ‘synthetic social support’–i.e. targeted forms of social network enhancement[6] to those within communities deemed at ‘high risk’ of developing both communicable and non-communicable diseases.

Early developments were in maternal health, but the use of community health workers has transferred to numerous other settings, including the management of non-communicable diseases in developed countries. Targeted prevention services, including using community health workers in high risk populations, have been seen as a cost effective way of making inroads to the problem, sometimes alongside medical interventions where they are available[4, 7–10]. Other trends have included engaging volunteers, ‘expert patients’ and other community roles–such as hairdressers or fire workers–in the wider public health workforce[11–13].

Despite the growing interest in community health work in both policy and research, there has been notably little consideration of how CHWs themselves understand, interpret and experience their role[14]. Nor have there been attempts to understand this experience within the wider social context of the new public health, within a risk society[15,16]. In this article, we analyse co-produced, situated accounts and photographic images of community health work from ‘health trainers’ (HTs), a type of CHW, in Sandwell, a deprived, ethnically diverse, post-industrial region of the Midlands, England, UK. The HT service was funded, free at the point of access, by the National Health Service (NHS) via outsourcing to not-for-profit, community interest companies[17].

## Background: Community health workers and health inequalities in England

Escalating costs of health care due to an ageing population and the related rise in non-communicable disease, such as cardiovascular disease (CVD), have led for political calls for new, more cost-effective approaches to prevention. CVD is the biggest cause of death in the world and is strongly associated with deprivation and other demographic factors, such as ethnicity[18]. The cost of cardiovascular disease to the UK economy, including health care, productivity loss and informal care costs, at the time of the introduction of health trainer services (2004) was estimated at £29.1 billion[19]. NHS health trainers emerged as a policy concept in 2004 and by 2008 concrete competencies had been developed for the role[20]. The White Paper *Choosing* Health[21] announced the launch of a ‘new kind of personal health resource’:

health trainers will be drawn from local communities […] will be accredited by the NHS to have skills appropriate for helping members of their community to achieve the changes that they want to make. In touch with the realities of the lives of the people they work with and with a shared stake in improving the health of the communities that they live in, health trainers will be friendly, approachable, understanding and supportive (DH, 2004: 104)

The white paper argued that partnership working (between NHS health improvement and community development services) and personalization of care (including tailoring public health messages) were central to success in enabling individuals to make healthy choices. Neither of these approaches was strongly supported by evidence. The ideology of individual (rather than state/collective) responsibility for health was long ago shown to be ineffective in tackling health inequalities due to failure to consider social causation of ill-health[22] and its correlate intervention of ‘health education’ had its own problems due to the unintended desensitising effects of multiple public health messages[23]. Public health and social science researchers have tended to argue that both individual and social/environment intervention is needed for change[24], as well as a consideration of lifecourse perspectives in terms of the accumulation of risk or disadvantage[25]. Drives for partnership working were also driven by political ideology and assumed to be effective rather than being supported by evidence from evaluations[26, 27].

Nevertheless, NHS health trainers were framed as a new tool to tackle the intractable[28] issue of health inequalities. The theory underpinning the use of CHWs in disease prevention and health promotion services has not always been well articulated[29] but it is generally agreed that a ‘one-size-fits-all’ approach should be avoided both in terms of the way the policy is implemented and the nature of the intervention[30, 31]. A contextual understanding of the barriers to lifestyle change offered by someone familiar with the local environment and community was considered likely to aid delivery[32]. While many commentators have argued that the intervention is unlikely to reduce health inequalities because of the failure to tackle the social determinants of health[20, 24, 33], there is some tentative evidence to suggest that CHW interventions are nonetheless effective at engaging ‘hard to reach’ populations for prevention such as younger, working people and men[34] and that certain approaches to implementation, such as targeted case finding, may enable a more efficient and cost-effective identification of those at higher risk of CVD[8, 9]. Carr et al.[4] have called for further investigation into the both the way that CHWs deliver tailored messages, and the nature and effectiveness of workers having a sense of ‘allegiance’ to the community they work with.

There have been some explorations of health trainer experiences which have noted aspects of the recruitment process, the backgrounds of health trainers and their training needs[31]. These accounts have noted challenging aspects of the role, such as the stress of engaging with clients with complex problems, the low pay and limited career options with the services leading them to seek ‘medicalized’ and/or professionalised roles elsewhere in the health service[35, 36], and the tensions between the adoption of a formal role in the community with their lay identities, potentially leading them to overemphasise their similarities to clients[14]. However, there has been little engagement theoretically with the tensions inherent in doing this kind of work, or its embodied impact. The purpose of this article is to understand more about how health trainers perceive their occupational identity both in relation to the health services and to their local communities, the role and purpose of their work, and its impact on them.

## Theoretical framework: Risk work or resilience work?

There is a useful theoretical distinction, which we interrogate in this article, between *risk*–the probability of an (adverse) event happening in the future–and *resilience*–the capacity to recover quickly from adversity or to succeed despite adversity. These concepts are often applied to describe the inherent character of communities (communities at risk, resilient communities) or individuals (person at risk, resilient person) and, in doing so, they tend to imply fundamentally different approaches–risk focuses on deficits or weaknesses that must be addressed, while resilience implies an asset-based approach that identifies strengths. When pairing these two concepts with the concept of ‘work’–everyday practices in paid (or unpaid) employment–as ‘risk work’ or ‘resilience work’ we can use them to explore and critique the practices and experiences of community health workers.

A ‘risk-based’ intervention is one where uncertain (client) futures are conceptualised through the lens of probabilistic accounts of the chances of an adverse event occurring. Risk has become an increasingly dominant framework in public services internationally[15, 37], including in the health services[16, 38–41]. The potential implications of a risk-based intervention for the nature of the work and the experience of the worker is that clinical interventions become purely technical, based on pre-calculated risk thresholds, that little ‘indeterminacy’[42] is involved, and that this would lead to reduced autonomy in the clinic and de-professionalization[43, 44].

A ‘resilience-building’ intervention is one that seeks to enhance coping and competence and, therefore, the ability to succeed or remain healthy despite a challenging environment. Much of the literature on resilience building has focused on individualistic, psychological interventions rather than social change[45] and this has been seen, by social scientists, as emblematic of late modernity and the individualization of society[46, 47]. Therefore, it may be that the differences between ‘risk’ and ‘resilience’ models as a basis for health promotion intervention have been exaggerated as they both often result in interventions targeted at producing individual-level behaviour change. Nevertheless, we do not know whether there is a difference between the two models in terms of the practices and experiences of the workers.

In this article, we take a critical approach and argue that it is important to look beyond the limitations of individualised models of risk and resilience. While, at present, they only represent a small literature, social scientific studies of ‘risk work’ and ‘resilience work’ provide a useful theoretical framing for these questions. They focus on how these forms of work ‘get done’–i.e. the practices[48, 49] and intersubjective, embodied experiences and identities of workers[50–53]. These approaches resist the notion of risk or resilience being intrinsic properties of individuals, but regard them as emergent properties of systems. In the case of UK health trainers, their work is often undertaken in a context of post-industrial communities characterised by high unemployment[54] and superdiversity[55].

What is additionally useful about both these approaches–risk work and resilience work–is that they focus our analytical attention on the tensions inherent in the work, ones that often remain ‘veiled’ as workers muddle through in an everyday sense[56], such as the mismatch between probabilistic knowledge about health risk and individual experiences of uncertainty[50].

## Methodology

### Design

These research questions emerged from previous empirical research on the implementation of a primary prevention programme[57] as part of an NIHR funded initiative–the Collaborations for Leadership in Applied Health Research and Care (CLAHRC). Themes of embodied worker experiences (Health Trainers) and tensions arose in this study, but we did not reach data saturation, so we set out to collect new empirical data to address these emergent questions. Our objective was to understand how community health workers experienced the tensions between biomedical and community based models of health promotion in their daily practice. We explore how these health trainers make sense of the health risks faced by the community that they live and/or work in and their own role within that community in relation to health promotion. Given the lack of previous literature about the subjectivity and meaning-making of practitioners undertaking risk/resilience work[50] the adoption of grounded theory[58] followed by abductive analysis[59] was appropriate. We use (and ultimately extend) the sensitizing theories of ‘risk work’ and ‘resilience work’ as a lens to analyse and critique the form of health promotion they are delivering and explore how they resolve in their embodied practices the tensions between biomedical, biopsychosocial and community development approaches to health promotion. We adopted an ‘situated interviewing’ approach[60] that allowed the voices and perceptions of the health trainers to come to the fore, yet enabled them to represent their experience of place within the local community in an explicit way, and then relate it back to their personal and occupational experiences. Photo-voice methodology[61] enabled both visual data (photographs taken by the participants) and oral data (collected in individual interviews) to be produced. The purpose was to co-construct with the participants a situated account of what it meant to them to be a health trainer in Sandwell. The core research team comprised NG (PhD), a White British female health sociologist born outside of the region but now living in Birmingham, who designed the project in collaboration with the service and MS (PhD), a male British Punjabi applied health service researcher with a background in sociology born and raised in Birmingham who conducted the interviews.

### Setting

Sandwell is a borough in a region called the Black Country (named so because of its industrial past) in the West Midlands of England, UK. The decline in UK industry has left the region one of the most deprived in the country, with life expectancy markedly less than the national average and residents at greater risk of developing long-term health conditions, such as CVD[62]. It comprises a number of small towns that have over time joined together into one conglomeration that sits on the edge of Birmingham, the second most populated city in the UK. The population is very ethnically diverse, including the white British population, established communities from migrants and descendants from Ireland, the 1960s mass migration from the Commonwealth (Black Caribbean, Punjabi Sikh, Mirpuri Muslim and Hindu Guajarati), as well as more recent immigrants from Eastern Europe, Africa and the Middle East[55, 63]. There is significant spatial segregation between ethnic groups as has been seen in other former industrial areas[64, 65]. However, Sandwell has been a beneficiary of significant investment and regeneration efforts–with regard to retail, education, transport and other infrastructure and housing–designed to address issues of employment, community cohesion and health inequalities[66–70].

### Intervention

At the time of data collection (August-September 2013), Sandwell primary care teams offered the health trainer service, via general practitioner (GP) referral, to individuals that were assessed as overweight, obese, and at high risk of developing vascular conditions such as diabetes or coronary heart disease. Health trainers were recruited from the local communities they served so they would have greater contextual and nuanced knowledge of the socio-cultural barriers faced by the population they treated. A key selection criteria was health trainers being chosen because they too had completed a significant lifestyle change and demonstrated skills of building and maintaining relationships with clients that would lead to behavioural change[56]. Training was varied and often needs based with many health trainers developing skills which focused more greatly on clinical aspects of their role. Health trainers were supervised by one designated manager whereby performance was monitored by meeting key performance indicators determined by the local healthcare provider which included: patient attendance to consultations, reduction in weight, reduction in blood pressure, and number of patients being referred to smoking cessation services.

Health trainers were embedded in GP surgeries and health centres across the borough, with the belief they would integrate into existing primary health care teams. It is important to note that, in line with trends more generally in the UK context to deliver community-based health services via not-for-profit organisations or social enterprises rather than the public sector (National Health Service), health trainers had moved from being NHS employees to being employed by a not-for-profit, community interest company[17]. Health trainers were equipped to: monitor blood pressure, take height and weight (to calculate body mass index), complete glucose testing, provide lifestyle, smoking cessation and weight management support, as well as encouraging increased physical activity. The implementation of health trainers varied nationally, but Sandwell was highly committed to recruiting health trainers from specific local areas and embedding them in practices within the vicinity[57].

### Access, recruitment & sampling

The wider CLAHRC research team had a long-established relationship with the primary care services in Sandwell and had been involved in designing the intervention[8, 9, 71]. NG had previously conducted qualitative interviews with some members of the health trainer team as part of a study evaluating the implementation of the prevention programme[57] and undertaken photo-voice interviews with patients who had been through the programme. The health trainers (who had helped recruit patients) expressed interest in the photo-voice method and so we worked with them to co-construct a project that focused on their experiences of working in the community. We attended a team meeting to provide information about the project and all currently employed health trainers and the manager were invited to participate. All but one (who did not give a reason) agreed to be involved.

### Data collection

Participants who had provided oral consent were given a disposable camera (or could take photos on their own digital cameras or mobile phones if preferred) and asked to take photos of anything that struck them as important for their own health and happiness or that of the communities that they worked with. The cameras or photos were sent back and processed and then an interview was scheduled. Written consent was taken before the commencement of the interview, which was digitally audio-recorded and completed at the participant’s place of work. The interviews, conducted by MS, were structured in two parts: the participants shared the photos one-by-one and discussed why they had taken images and the interviewer used probes to draw out full explanations. Typically, photographs can have multiple meanings: in most cases, the photos shared had either ‘concrete/literal’ or with ‘first order’ (using culturally familiar analogies), rather than more complex or hidden ‘second order’ meanings that required extensive explanation[72]. However, the common sense assumptions the researchers may have made about the ‘content’ of the image were drawn out through discussion, and sparked new lines of enquiry in the interview. At the end of the interview, the participants were asked to name someone who they considered healthy and to explain why (inspired by the method used by Blaxter[73] in her seminal study, *Health and Lifestyles*). Each interview lasted approximately 60 minutes.

### Data management and analysis

The photographs were broadly categorised by subject and the interviews were transcribed *verbatim* by a professional transcribing company. Initial coding of the transcripts was conducted independently by the authors who met to discuss the emerging themes and to agree on an analytic framework. Data were coded using N-Vivo. Initial descriptive analyses were conducted of the key themes to emerge from the study. These were fed back to the health trainers at a team meeting for a member check (using posters for each theme, populated with photos taken as part of the study). At this point, further theoretical reflection and re-engagement with the data using abductive reasoning with our two sensitizing concepts of risk work and resilience work[74] led to the development of three emergent themes: health spaces, occupational identity and embodiment–that we examined in terms of the emergent practices related to each–and a key cross-cutting theme around how being part of the community influenced the experience and practice of being a health trainer, although we noted that assumptions of the similarities between lay workers and their target population have been questioned[14]. As part of our analysis and interpretation, the authors presented initial descriptive themes using posters and oral presentation showcasing findings to health trainers. This gave our participants the opportunity to learn, ask questions and further refine the authors’ interpretations and confirm data saturation. Final interpretative analysis was conducted by the authors alone.

We report the data anonymised but with detail on whether the health trainer was ‘raised’ (grew up as a child), ‘lived’ (moved to the area as an adult), or ‘worked’ (never lived) in Sandwell (see Table 1 for a summary of the participants). Ethical review and approval was obtained from the University of Birmingham Life and Health Sciences Ethical Review Committee. Written consent was obtained from all participants to be interviewed and to use their photos, and data are reported anonymously.

**Table 1:** Participant characteristics.

Participant characteristics		Number (Total = 11)
Experience of the local community		
	Raised, living and working in Sandwell	3
	Living and working in Sandwell (raised elsewhere)	4
	Working in Sandwell (raised and living elsewhere)	4
Gender		
	Male	5
	Female	6
Age		
	26–35	6
	36–45	2
	46–55	3
Ethnicity		
	White British	8
	British Pakistani	1
	Black African	1
	Indian Guajarati	1

## Findings

The photos that the participants took included scenes from both from their private spaces (homes and gardens), work spaces (office, equipment and buildings) and public spaces (streetscapes, public buildings and green spaces). Participants noted the significance of each photo variously in terms of physical, mental, social and emotional and spiritual health. They described the subject of their photos as things like ‘family’, ‘walking the dog’, ‘take away’, ‘kitchen’, ‘regeneration’, ‘planning’, ‘transport’, ‘fast food outlets’, ‘mother-in-law’s cooking’, ‘gardening’, ‘shopping’ as well as photos of key landmarks in Sandwell such as the local college, parks, the art gallery and the new supermarket/retail complex. These photos and the participants’ accounts of why they had selected them told a story of what it meant to be part of the community and to be involved in changing health practices within that community to reduce the risk of CVD.

### Being critical insider observers of the community’s health spaces

**Fig 1:**
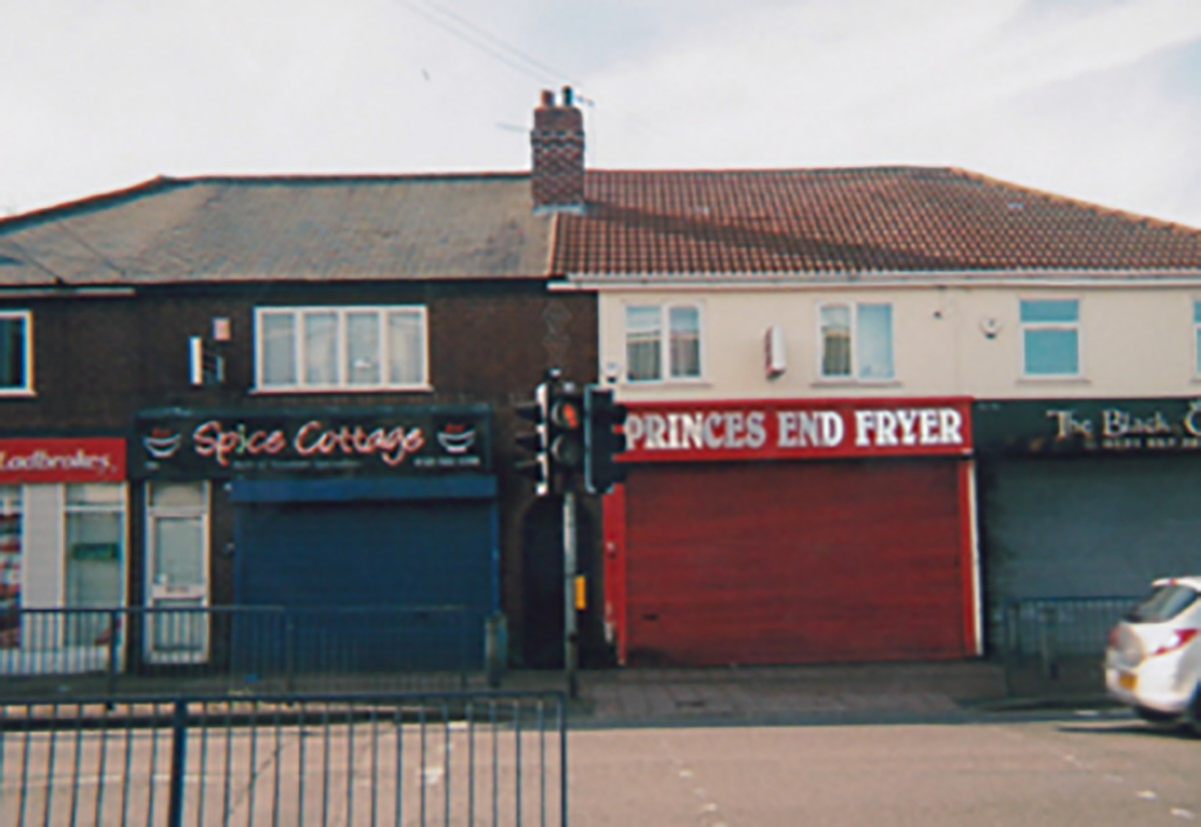
A local street scene with three fast food restaurants and a gambling shop.

**Fig 2:**
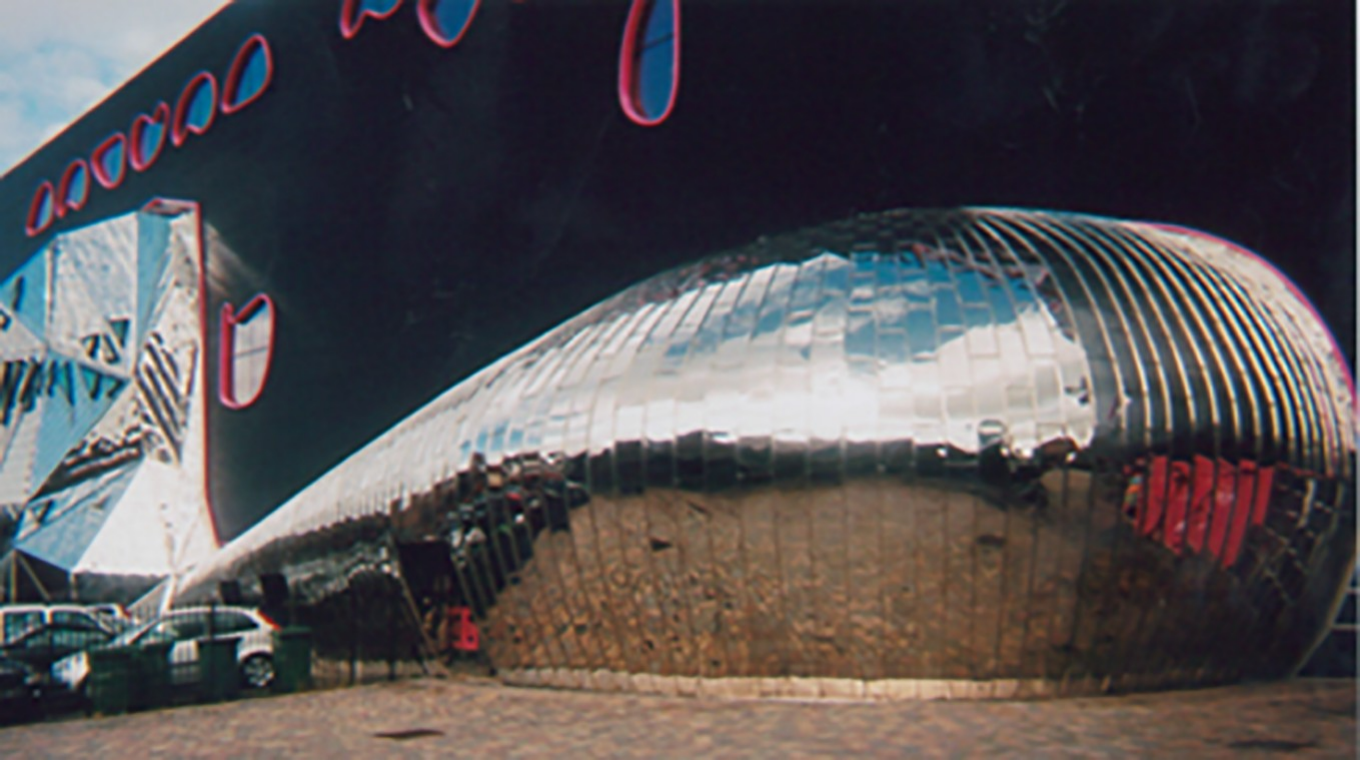
A local art gallery built as part of the regional regeneration.

The photographic images and accounts constructed by the health trainers about their work were full of commentary on the material and social environment in which their clients were living (e.g. Fig 1), with particular focus given to family, green/recreational spaces, planning and regeneration (e.g. Fig 2). Health trainers demonstrated in their accounts of these community spaces that they were *critical insider observers* of their local area, demonstrating keen awareness of the social determinants of health and of the constraints experienced by their clients in trying to change their ‘lifestyles’ to reduce their health risk:

And I think that’s what we struggle with in our job roles. We’re trying to teach somebody something in an environment where there’s that many barriers, which you will see in the pictures (HT7_raised)

The notion of being an insider–of understanding what life was like for people in Sandwell–was crucial to the accounts the health trainers’ gave of their working practices and this demonstrates that using concepts of risk or resilience as purely individual attributes is insufficient theoretically to interpret the nature of their work. There was nonetheless some variation in the accounts between those that tended towards asset-based community development and resilience models and those that embraced a more biomedical risk-based model of health promotion.

In the former, the health trainers often had a background in community-based work: one had worked as an activist around disability issues and around supporting women in business (HT4_working); another had previously worked as a youth worker in the area (HT3_raised), and another volunteered in a community gym training junior weightlifters (HT9_raised). HT2’s father had been a trustee of a local mosque and ‘my passion really grew for working in the community through my dad’; he noted that many of the younger generation were moving out of the area but that ‘*sometimes you need to use your experiences and whatever you’re learning and stay in your area to help that area’* (HT2_raised). These participants often saw their current role in very positive terms:

Every time I walk down the canal, at 8 o’clock in the morning, you’ve got ten people standing there with cans, my age [40s], who I went to school with and because industry in the Black Country has gone, it’s moved out, and these people have no academic skills, so what they’re turning to is chat, drink, smoke and they’re in a pit of despair […] I mean, me and you [MS], we work because of pride and respect (HT7_raised)

Others adopted a more biomedical and risk-framed notion of their occupational identity. They perceived their role though more traditional medical ideals: *‘I try to be approachable and understanding*. *It’s just a normal and professional relationship’* (HT6_working). They increasingly identified with the ‘medical/technical’ aspects of the role (locally often referred to ‘skills escalation’) and used terms such as ‘patient-centred care’ which were embedded in medical rhetoric. These skills and approaches were perceived to be given more value externally by health professionals, and linked this to their aspirations for progression within the context of NHS hierarches of professional roles:

I think the most part, the most important part of the health trainer role which I enjoy is the CVD screening because it really, really openings people’s eyes when you show them the risk scoring, you show them how to bring it down and I find it really rewarding (HT10_living).

### Delivering small but sustainable health improvements

Within this context of deprivation, disadvantage and the associated poor health outcomes, the health trainers saw themselves as trying to enable clients to unhook themselves from a cycle of poor health, through supporting them to make small but sustainable changes in their lives that would reduce their cardiovascular risk and increase their resilience.

An imbalance of 50 calories here or there can lead to an annual surplus of 1,800 calories or half a stone of body fat in 12 months. So those couple of Quality Streets or those couple of extra biscuits, it’s the difference between getting people to put weight on or lose weight. That’s what I really try to get over to the people that we see. I’m not looking to put them on some regimentally strict diet, I’m looking to pinch 50 calories from here or there and do this over a longer period of time’ (HT9_raised)

As this quote shows, in some ways, the health trainers uncritically accepted the biomedical principles of energy balance (less calories, more activity), but in addition to simply conveying that risk information to their clients, they also took three crucial additional steps–(i) tailoring to the individual in front of them (ii) working towards sustainable change and (iii) re-evaluating the process of applying the risk information to individuals over time.

**Fig 3:**
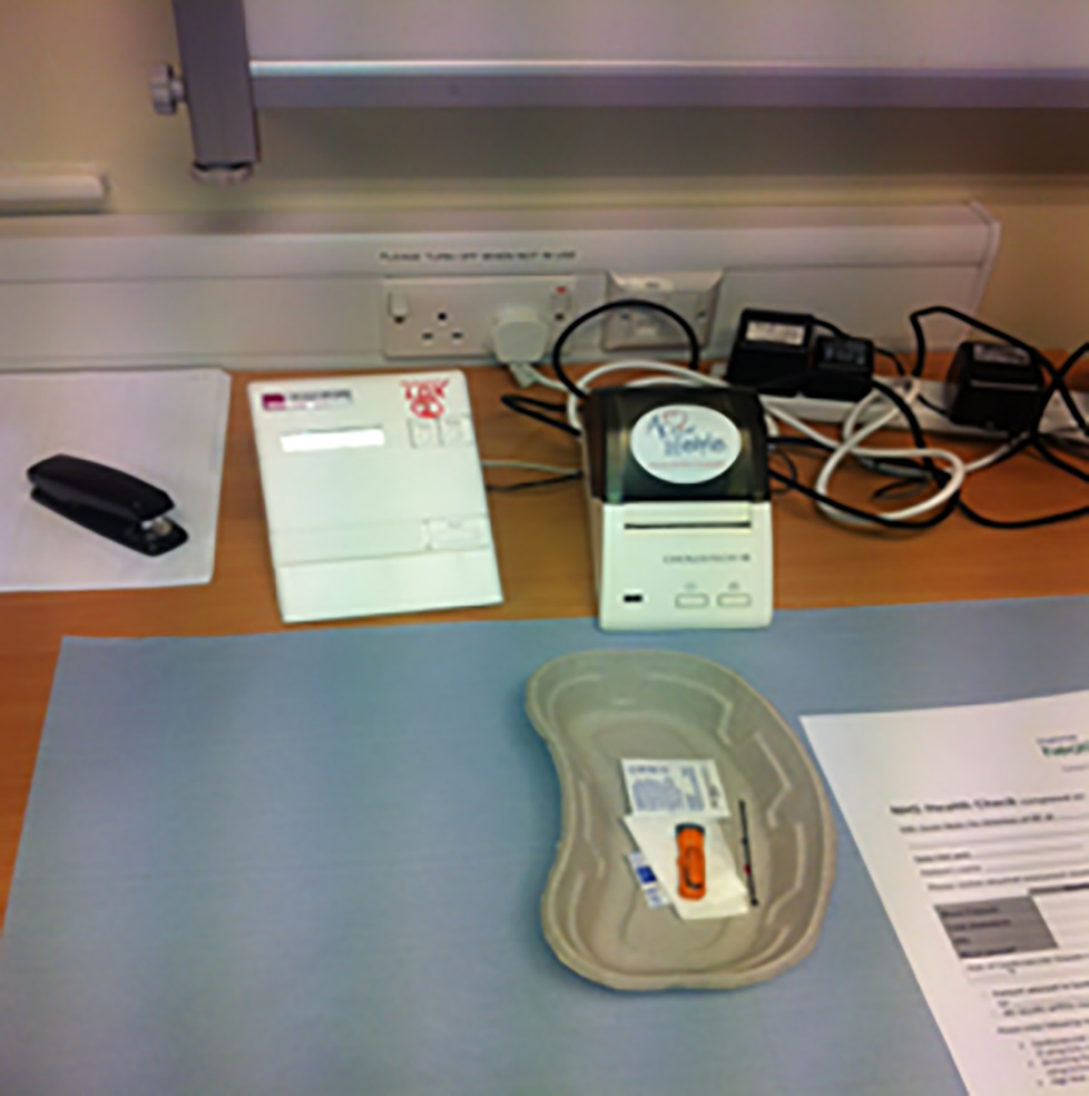
Health trainer desk showing forms, computer and medical technologies that they use on a daily basis.

**Fig 4:**
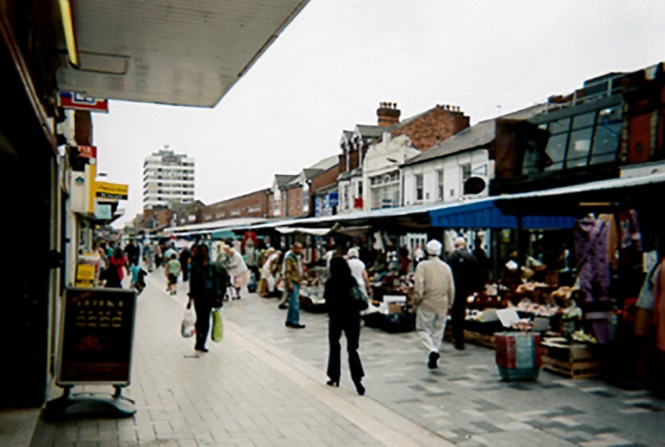
Image of the local high street market selling food and other items.

Many of the skills involved in the first step—tailoring—reinforce other findings in the literature, such as explaining ‘*what it is to live healthily in a simple way’* (HT6_working) and non-judgemental listening and ‘*listening to the stuff they don’t say’* (HT5_working). While the number generated through a formal CVD risk assessment was seen as ‘the risk’ (e.g. Fig 3), health trainers listened to clients to understand the wider context of their lives, including the other (non-health) risks they may be trying to balance (such as the safety of green spaces, or the affordability of food). The impact of these wider risks on the local population were complex, particularly for the diverse communities they were working with (e.g. Fig 4), facing challenging issues of employment, childcare, education and access to health care, and not within the remit of the CHW to measure or address. Nevertheless, the informal assessment of non-health risks was a precursor to negotiating and proceeding with the client, to understand the context in which they might be trying to implement lifestyle changes and helping to build resilience in this context.

Being able to create sustainable change is reliant on health trainers’ explicit or implicit appreciation the constraining and enabling effects of wider social networks and discourses in which their clients are embedded is much closer to definitions of resilience and less often articulated in the literature. In terms of embodied practices, this could mean acknowledging material circumstances but also exploring assumptions and habits: ‘*people say to me all the time*, *‘it’s really hard buying food because there’s only fast food shops*, *there’s no green grocers* … *and I don’t drive … so you really have to open that up’* (HT5_working). It could also mean intervening in the resilience of the wider community, such as working with community volunteers to lead walking groups (a number of the HTs did this).

### Practising what we preach

There remains an unresolved tension between the findings in the previous two sections–of the persistence of the social determinants of health and the health trainers’ role to try to initiate change in embedded health practices. While it could be framed simply in terms of an attempt to generate ‘resilience’ within communities to mitigate the negative effects of deprivation, our findings suggest that there is a crucial additional practice at play. In contrast to public health messages which are given directly to the target population though advertisements or other media, community health workers literally embody a mediating role, thereby making the messages more convincing. Being an accessible role model for healthy practices including being able to share personal experience, speaking local languages or dialects, or simply being able to speak in lay terms (Plain English):

So, from eating pies, chips and all the trimmings, I switched to tuna, all grain foods and lots of veg. And again, that’s where my passion came from for the lifestyle … I think I get a good result for the same reason I can identify, and they’ll say ‘Oh you’re one of these who don’t put weight on. You’ve never smoked’. I say ‘No, actually I’ve done it all’, and they’ll go, ‘No!’. [I say] ‘I’ve been where you are’. People can really, you know, lower the barriers then and they can start to tell you, but I think doctors approach sometimes may be, ‘You need to lose weight because if not you’re gonna have this and you’re gonna have’. I don’t think people wanna hear that. They know anyway. They know they should be eating this and that but you’ve gotta find out why. (HT7_raised)

**Fig 5:**
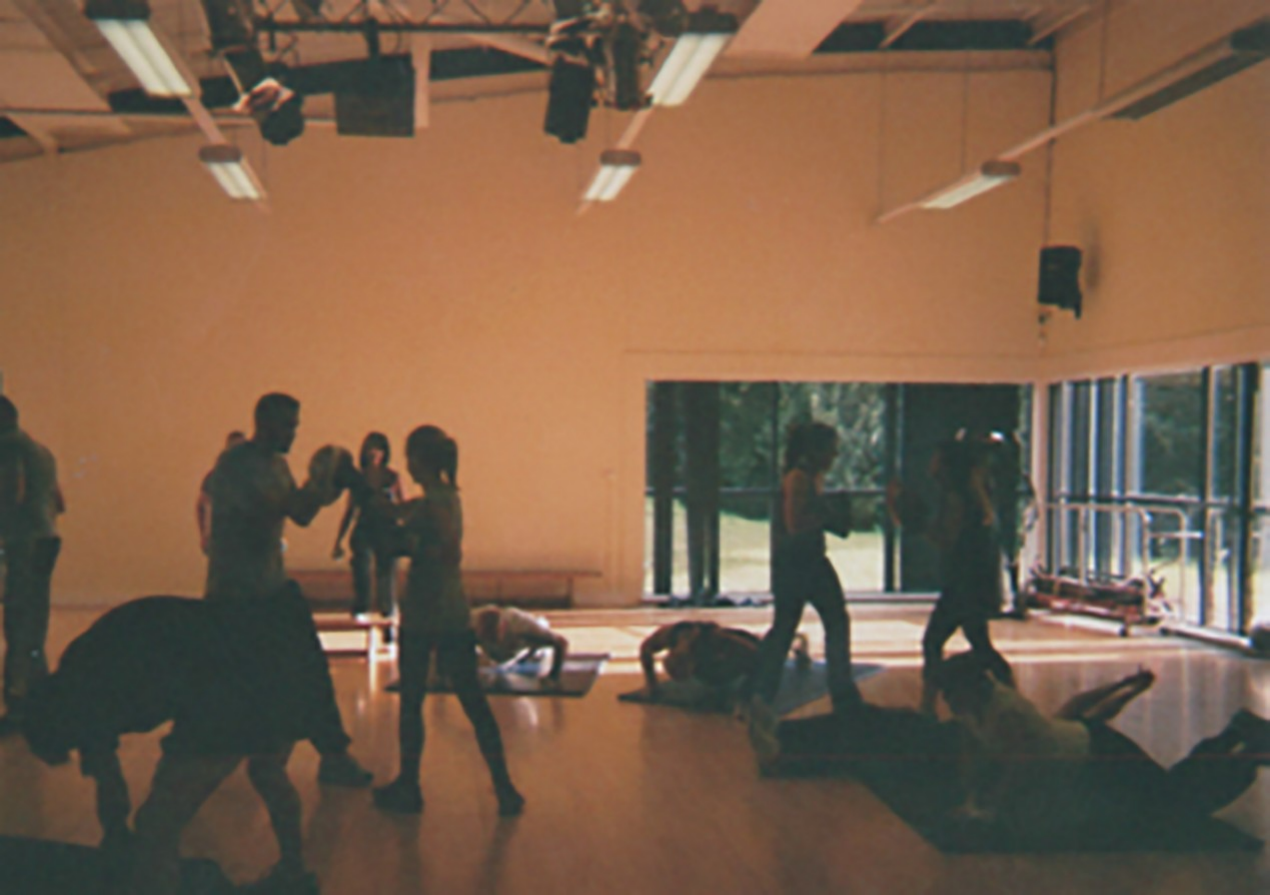
A local gym.

**Fig 6:**
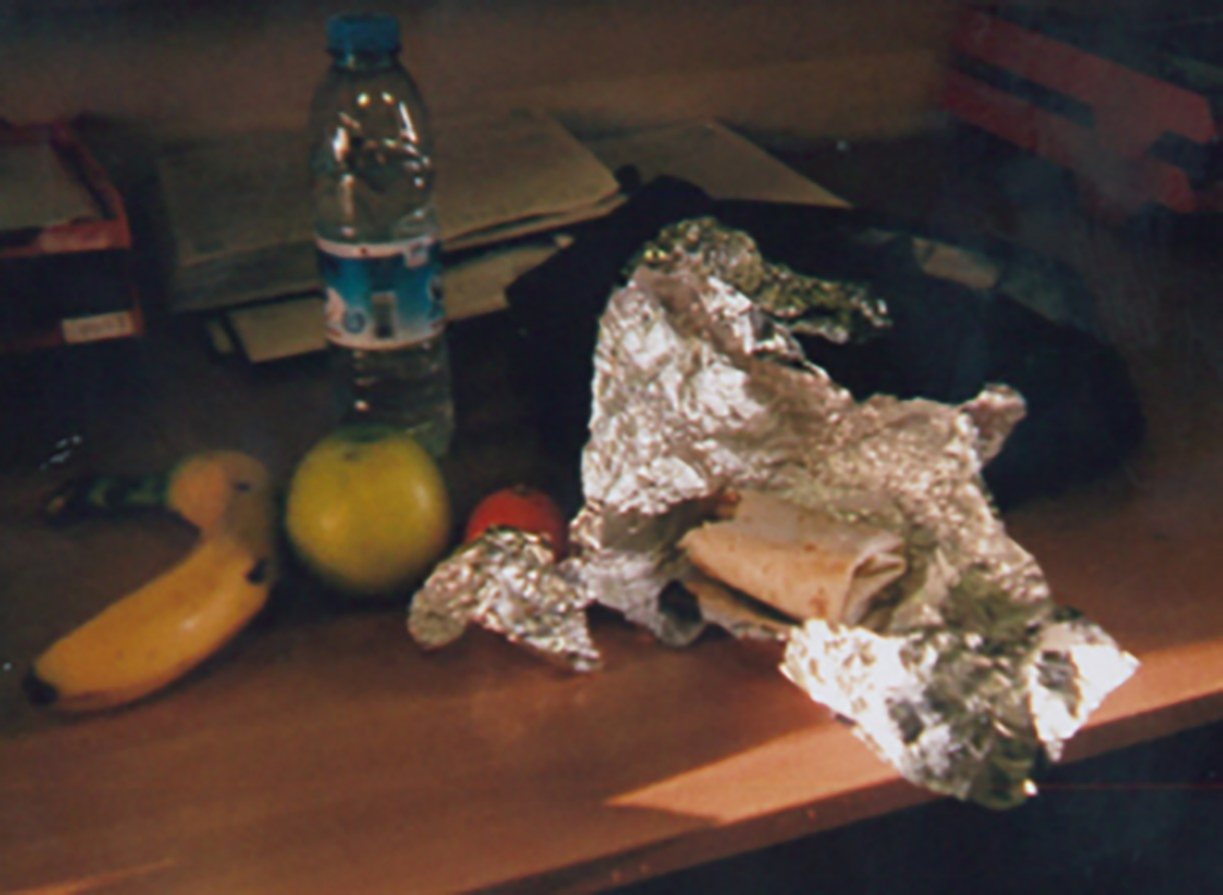
A health trainers’ packed lunch.

This should not be seen as a fundamental change in policy because it is still focused on individual behaviour change, but it is a new medium of delivering the message. The health trainer is the living embodiment of the public health message; in other words, they perform resilience in a constraining social environment. Many of the photos shared and the accounts given by the participants demonstrated their own personal commitment to a ‘healthy lifestyle’, though food and physical activity (e.g. Figs 5 and 6). They also noted the importance of employment, family and connections to nature or spirituality for health and happiness. It was even noted that the experience of transforming your life was part of what was needed to do the job:

I’ll hold my hands up, because I didn’t get the job at first, and I was basing my merit on the way I looked at the time, because I was in good shape […] and I couldn’t understand why some people went through as a smoker and a bit overweight […] and now I know. Because their journey–that person, yeah was still smoking but he’d come down from 40 to 10 and he was packing it in. That person was overweight, but she’d got thyroid problems […] all these people on the journey have also improved their health as health trainers, and we’ve learned from one another and still learn (HT7_raised).

This normative dimension–it being ‘good’ to be able to be a role model for resilience–highlights how fundamental this final, third, step is in the ability to accomplish community health work in the context of the dominance of risk logics in health services and to be able to manage the tensions between risk and the acknowledgement of the persistence of the social determinants of health.

## Discussion

Our findings suggest that the differences between risk-based and resilience-based approaches to health promotion may have been exaggerated as both concepts have the potential to be deployed in ways that reinforce and sustain modernist, neo-liberal ideologies–focusing on the individual, rather than the social[15, 45]. By focusing on the practices and subjectivities of health trainers, a type of community health worker, we were able to show that risk-based and resilience-based models of health promotion are not necessarily diametrically opposed but share important features, especially when the focus is on risk and resilience as ‘characteristics’ of individuals or communities. However, we also have shown that the health trainers did not view their work purely in terms of individual intervention. Ideas of risk and resilience in their accounts and images were deeply embedded in families, communities and the local political environment, and they showed a keen understanding of the social determinants of health[75, 76].

Our study has offered a new perspective on the contribution of health promotion interventions for prevention of disease–and particularly help unravel the complex relationship between biomedical and community based forms of intervention. Whereas many of the studies described in the background focus on outcomes or methods, our focus on the embodied experiences of those delivering the intervention brings new insights to how policies that draw on logics of ‘risk’ and theories of ‘resilience’ are operationalised at the street-level. Risk is a hard concept to grasp[77] and it is ‘partly through risk work that risk is made socially and politically compelling to those “at risk”[50]. The intervention that the health trainers were part of–CVD risk assessment followed by lifestyle interventions—was originally framed (and funded) around biomedical concepts of ‘risk’ reduction, but in the process of its implementation it changed[6, 78]. The ‘client-facing’ practitioners were also committed to caring for their clients in the context of attempting to reduce risk(s)[50, 79] which resulted in a much more complex picture and one in which they described intervening with clients in ways that can be conceptualised as resilient moves[48]. Key to Aranda and Hart’s theoretical perspectives is that ‘resilient moves’ are embodied and emergent practices rather than an inherent quality of an individual; by thinking in this way, they open up the possibility to think of ‘a resilient move *as an intervention* that can tinker with injustice by disturbing, converging or transforming other practices’ (Aranda and Hart, 2015:361, our emphasis). Hence, the health trainer is the living embodiment of public health behavioural change messages; as Aranda, Zeeman, Scholes and Morales[80] explain, ‘The resilient subject becomes someone who at any given time, across their biography or lifespan identifies or misidentifies in complex ways with demands to be resilient’ (Aranda et al., 2012: 555).

Health trainers were able to negotiate the tension between epidemiological knowledge about health risks and the individual, contextualized experiences of their clients, through adopting a critical insider perspective on health risks in the community, and recognising the constraints on individual lifestyle choices facing the community. In this context, health trainers took pride in their occupation which was framed by policy makers as minimizing CVD risk but, in its implementation, shared many features of community-based resilience work. The health trainers saw themselves supporting their clients to make small but significant changes to improve their health, despite the constraints of the social environment and enabling resilient moves in the face of this. As Mol, Moser and Pols^[^^79^^]^ argue, the success of medical technologies depends ‘on people willing to adapt their tools to a specific situation while adapting the situation to the tools, on and on, endlessly tinkering’. Finally, the health trainers attempted to resolve any tensions in their relationships with clients that might emerge as a result of the uncertainty of what CVD ‘risk’ might mean for a client’s health future, by embodying their own health advice, performing resilience, and therefore able to share their experiences with clients, thereby making it easier for clients to identify with and trust them.

The methodological strengths of the study were that: all but one of the health trainers working in the region participated and they were made up of a diverse group (by ethnicity, age, gender, migration status and socio-economic background); the application of the photo-voice methodology was successful for the facilitation of place-based talk, building narratives of health and place, and giving interviewees greater autonomy and scope in co-producing the data with the interviewer; and the involvement of an interviewer born and raised locally, who therefore had contextualised knowledge and understanding of the area. However, the study also had limitations as it was based on data from one type of community health worker—UK health trainers—and in just one part of the UK, and therefore other CHWs may have different experiences. The substantive findings are most likely to be transferable to other similar post-industrial suburban areas where recruitment of CHWs was conducted in a similar way to ensure local people were employed. Indeed, the data collected in this study reinforced and extended previous findings about the experience, careers and challenges for health trainers in other parts of the country[14, 34, 81, 82].

## Policy implications

From a policy perspective, the CHWs in this case were able to be critical of certain aspects of community life that were beyond their control, such as urban planning and regeneration or socio-economic deprivation. However, they remained uncritical in their acceptance of the translation of epidemiological science to the lifestyle interventions they were delivering. Their own personal histories, identities and practices were bound up in the idea of a ‘healthy lifestyle’ thereby making perhaps the ‘perfect’ vessel for health education messages, as living proof of their effectiveness. Their work was deemed of only limited value to the system which continued to grade the roles at a very low level and with limited progression opportunities. Both recruitment and retention of CHWs are challenging issues for policy makers.

The delivery of CHW services in the UK is now largely through outsourcing to non-profit making, community interest companies (CICs). The recruitment of CHWs is mostly completed by the CICs, and although commissioners may insist on certain features in the job description, CICs are given considerable freedom in their approach. The recruitment process varies considerably nationally, and the findings from this paper gives insights about the extent to which an understanding of the local community should be important, as well as the more technical/administrative tasks of undertaking risk assessments. The recruitment of CHWs relies on applicants bringing with them certain knowledge and skills, so that rather than needing to be trained on the wider context of (non-health) risks, soft skills in communication, local knowledge of the environment and activities available, they have this already. The formal training most receive would be around how to apply risk assessment tools, facilitating behavioural change, or the health promotion and education messages they are expected to deliver.

Where CHWs are often embedded in primary care (GP practices), such as in the case of health trainers, the outsourcing of the service from the NHS can seem paradoxical making it difficult for healthcare professionals and clients alike to interpret their role. In the case of health trainers, outsourcing the services from the NHS has also increased levels of job insecurity as their employment was dependent on securing contracts to deliver the service. However, not all CHWs have a role in primary care, many are much more community based. The retention of CHWs remains problematic, notably due to low pay, job insecurity, the emotionally and practically intensive nature of the role, and their poorly recognised para-professional status in a professionalised health system. The skills escalation model is limited and based on a rigid medical conception of ‘professionalized’ roles. While the CHW tasks are acknowledged by CIC managers, health professionals and policy makers, their value in terms of medical ‘effectiveness’ is continually under question as these interventions do not necessarily result in reductions in health inequalities nor directly in clinical outcomes.

## Conclusion

We have interrogated the ways in which CHWs experience of belonging to the ‘high risk’ communities with which they are working, shapes occupational identity and everyday working practices. Exploring the tensions in the role through the lens of theories of risk work and resilience work, that emphasise embodied practices, has enabled us to make three important theoretical steps for understand how health promotion activities are carried out by CHWs in their own communities: first, that being a *critical insider* enables CHWs to make sense of the diverse constraints on lifestyles within their community; second, that they understand their own role as constrained by operating within this context so that they aim to support clients to make *small but sustainable* changes to their lifestyles, and third, that the uncertainties and tensions of translating population based risk information to individual clients is (at least partially) resolved at an embodied level, with the CHWs becoming accessible *role models* for the value of changed lifestyles. Our focus on the embodied practices of those delivering the intervention is an original contribution to the literature and enables a much more nuanced explanation for the intervention’s successes in engaging communities in new ways with health promotion, and its limitations in terms of failing to address the underlying causes of health inequalities. While these sorts of intervention constitute a new mode of delivery of health information, it is not a fundamental theoretical or political shift away from individual/behaviour interventions towards more socio-structural approaches to tackling health inequalities.

## Supporting information

S1 File Highlights. (DOCX)

S2 File COREQ (COnsolidated criteria for REporting Qualitative research) checklist. (PDF)

## References

[1] Cairns J.M., Curtis S.E., Bambra C., 2012 Defying deprivation: A cross-sectional analysis of area level health resilience in England. Health & place 18, 928–933. 22440779 10.1016/j.healthplace.2012.02.011

[2] Doran T., Drever F., Whitehead M., 2006 Health underachievement and overachievement in English local authorities. Journal of Epidemiology & Community Health 60, 686–693. 16840758 10.1136/jech.2005.041889 PMC2588077

[3] Tunstall H., Mitchell R., Gibbs J., Platt S., Dorling D., 2007 Is economic adversity always a killer? Disadvantaged areas with relatively low mortality rates. Journal of Epidemiology & Community Health 61, 337–343. 17372295 10.1136/jech.2006.049890 PMC2652946

[4] Carr S.M., Lhussier M., Forster N., Geddes L., Deane K., Pennington M., Visram S., White M., Michie S., Donaldson C., Hildreth A., 2011 An evidence synthesis of qualitative and quantitative research on component intervention techniques, effectiveness, cost-effectiveness, equity and acceptability of different versions of health-related lifestyle advisor role in improving health. Health Technol Assess 15, iii–iv, 1–284. 10.3310/hta15090 PMC4781312 21329611

[5] Singh P., Chokshi D.A., 2013 Community health workers—a local solution to a global problem. New England Journal of Medicine 369, 894–896. 10.1056/NEJMp1305636 24004115

[6] Gale N. K., Kenyon S., MacArthur C., Jolly K., & Hope L. (2018). Synthetic social support: Theorizing lay health worker interventions. Social Science & Medicine, 196, 96–105. 10.1016/j.socscimed.2017.11.012 29169057

[7] Barton G.R., Goodall M., Bower P., Woolf S., Capewell S., Gabbay M.B., 2012 Increasing heart-health lifestyles in deprived communities: economic evaluation of lay health trainers. Journal of Evaluation in Clinical Practice 18, 835–840. 10.1111/j.1365-2753.2011.01686.x 21518152

[8] Crossan C., Lord J., Ryan R., Nherera L., Marshall T., 2017 Cost effectiveness of case-finding strategies for drug treatment in primary prevention of cardiovascular disease: a modelling study. British Journal of General Practice 10.3399/bjgp16XXXXXXX PMC5198616 27821671

[9] Hemming K., Ryan R., Gill P., Westerby P., Jolly K., Marshall T., 2016 Targeted case finding in the prevention of cardiovascular disease: a stepped wedge cluster randomised controlled trial. Br J Gen Pract, bjgpoct-2016-2066-2651-marshall-fl-p. 10.3399/bjgp16X686629 PMC5033312 27528707

[10] Marshall T., 2010 Targeted case finding for cardiovascular prevention. BMJ 340. 10.1136/bmj.c1376 20418544

[11] Lorig K., 2002 Partnerships between expert patients and physicians. The Lancet 359, 814–815. 10.1016/S0140-6736(02)07959-X 11897275

[12] RSPH, 2015 Rethinking the Public Health Workforce. Royal Society for Public Health, London.

[13] Taylor D., Bury M., 2007 Chronic illness, expert patients and care transition. Sociology of health & illness 29, 27–45. 17286704 10.1111/j.1467-9566.2007.00516.x

[14] Cook T., Wills J., 2012 Engaging with marginalized communities: the experiences of London health trainers. Perspectives in Public Health 132, 221–227. 10.1177/1757913910393864 22991369

[15] Beck U., 1992 Risk society: Towards a new modernity. Sage, London.

[16] Petersen A., Lupton D., 1996 The new public health: Health and self in the age of risk Sage Publications, Inc.

[17] Rees J., Mullins D., 2016 The third sector delivering public services: Development, innovations and challenges Policy Press, Bristol.

[18] DH, 2008 Putting Prevention First—Vascular Checks: Risk Assessment and Management. Department of Health, London.

[19] Luengo-Fernández R., Leal J., Gray A., Petersen S., Rayner M., 2006 Cost of cardiovascular diseases in the United Kingdom. Heart 92, 1384–1389. 10.1136/hrt.2005.072173 16702172 PMC1861058

[20] Martindale A.-M., 2013 The UK Health Trainer Initiative: Critical Contexts, in: Petrie S. (Ed.), Controversies in Policy Research: Critical Analysis for a New Era of Austerity adn Privation. Palgrave Macmillan, London, pp. 159–178.

[21] DH, 2004 Choosing health: Making healthy choices easier Department of Health, London.

[22] Crawford R., 1977 You are Dangerous to Your Health: The Ideology and Politics of Victim Blaming. International journal of health services 7, 663–680. 10.2190/YU77-T7B1-EN9X-G0PN 410739

[23] Davison C., Smith G.D., Frankel S., 1991 Lay epidemiology and the prevention paradox: the implications of coronary candidacy for health education. Sociology of health & illness 13, 1–19.

[24] Trayers T., Lawlor D.A., 2007 Bridging the gap in health inequalities with the help of health trainers: a realistic task in hostile environments? A short report for debate. Journal of Public Health 29, 218–221. 10.1093/pubmed/fdm046 17636301

[25] Graham H., 2002 Building an inter-disciplinary science of health inequalities: the example of lifecourse research. Social Science & Medicine 55, 2005–2016. 10.1016/s0277-9536(01)00343-4 12406467

[26] Blackman T., Harrington B., Elliott E., Greene A., Hunter D.J., Marks L., McKee L., Williams G., 2012 Framing health inequalities for local intervention: comparative case studies. Sociology of health & illness 34, 49–63. 21668455 10.1111/j.1467-9566.2011.01362.x

[27] Perkins N., Smith K., Hunter D.J., Bambra C., Joyce K., 2010 'What counts is what works'? New Labour and partnerships in public health. Policy & Politics 38, 101–117.

[28] Exworthy M., Blane D., Marmot M., 2003 Tackling Health Inequalities in the United Kingdom: The Progress and Pitfalls of Policy. Health Services Research 38, 1905–1922. 10.1111/j.1475-6773.2003.00208.x 14727803 PMC1360979

[29] Turner G., Shepherd J., 1999 A method in search of a theory: peer education and health promotion. Health Education Research 14, 235–247. 10.1093/her/14.2.235 10387503

[30] Releford B.J., Frencher S.K., Yancey A.K., Norris K., 2010 Cardiovascular Disease Control Through Barbershops: Design of a Nationwide Outreach Program. Journal of the National Medical Association 102, 336–345. 10.1016/s0027-9684(15)30606-4 20437741 PMC3758504

[31] Visram S., Clarke C., White M., 2014 Making and Maintaining Lifestyle Changes with the Support of a Lay Health Advisor: Longitudinal Qualitative Study of Health Trainer Services in Northern England. PLoS ONE 9, e94749 10.1371/journal.pone.0094749 24801173 PMC4011706

[32] Luque J.S., Ross L., Gwede C.K., 2014 Qualitative Systematic Review of Barber-Administered Health Education, Promotion, Screening and Outreach Programs in African-American Communities. Journal of Community Health 39, 181–190. 10.1007/s10900-013-9744-3 23913106 PMC3947222

[33] Smith J., Gardner B., Michie S., 2010 Health Trainers national end of year report: 2008–09. London: University College London.

[34] Visram S., Carr S.M., Geddes L., 2015 Can lay health trainers increase uptake of NHS Health Checks in hard-to-reach populations? A mixed-method pilot evaluation. Journal of Public Health 37, 226–233. 10.1093/pubmed/fdu041 24990955 PMC5942531

[35] Brown C., Hennings J., Caress A.-L., Partridge M., 2007 Lay educators in asthma self management: Reflections on their training and experiences. Patient education and counseling 68, 131–138. 10.1016/j.pec.2007.05.009 17662568

[36] Mathers J., Parry J., 2013 A REVIEW OF THE IMPLEMENTATION OF THE NATIONAL HEALTH TRAINER SERVICE INITIATIVE (Final Report to Policy Research Programme, NIHR) School of Health and Population Sciences, University of Birmingham Birmingham.

[37] Kemshall H., 2002 Risk, Social Policy and Welfare. Open University Press, Buckingham.

[38] Flynn R., 2002 Clinical governance and governmentality. Health, risk & society 4, 155–173.

[39] Kemshall H., 2010 Risk rationalities in contemporary social work policy and practice. British Journal of Social Work 40, 1247–1262.

[40] Lupton D., 1993 Risk as moral danger: the social and political functions of risk discourse in public health. International journal of health services 23, 425–435. 10.2190/16AY-E2GC-DFLD-51X2 8375947

[41] Power M., 2004 The Risk Management of Everything: Rethinking the Politics of Uncertainty. Demos, London.

[42] Jamous H., Peloille B., 1970 Professions or self-perpetuating systems? Changes in the French university-hospital system, in: Jackson J.A. (Ed.), Professions and Professionalization. Cambridge University Press, Cambridge, pp. 111–152.

[43] Haug M.R., 1972 Deprofessionalization: an alternate hypothesis for the future. The Sociological Review 20, 195–211.

[44] Starr P., 2009 Professionalization and Public Health: Historical Legacies, Continuing Dilemmas. Journal of Public Health Management and Practice 15, S26–S30. 10.1097/PHH.0b013e3181af0a95 19829224

[45] Bottrell D., 2009 Understanding ‘marginal’perspectives: Towards a social theory of resilience. Qualitative Social Work 8, 321–339.

[46] Giddens A., 1991 Modernity and self-identity: Self and society in the late modern age Stanford University Press, CA.

[47] Rose N., 2001 The politics of life itself. Theory, culture & society 18, 1–30.

[48] Aranda K., Hart A., 2015 Resilient moves: Tinkering with practice theory to generate new ways of thinking about using resilience. Health: 19, 355–371. 10.1177/1363459314554318 25331646

[49] Horlick-Jones T., 2005 On ‘risk work’: professional discourse, accountability, and everyday action. Health, risk & society 7, 293–307.

[50] Gale N. K., Thomas G. M., Thwaites R., Greenfield S., & Brown P. (2016). Towards a sociology of risk work: A narrative review and synthesis. Sociology Compass, 10(11), 1046–1071.

[51] Nading A.M., 2013 Love Isn't There in Your Stomach. Medical anthropology quarterly 27, 84–102. 10.1111/maq.12017 23674324

[52] Scamell M., 2011 The swan effect in midwifery talk and practice: a tension between normality and the language of risk. Sociology of health & illness 33, 987–1001. 21668457 10.1111/j.1467-9566.2011.01366.x

[53] Warner J., 2006 Inquiry reports as active texts and their function in relation to professional practice in mental health. Health, risk & society 8, 223–237.

[54] Bambra C., 2010 Yesterday once more? Unemployment and health in the 21st century. Journal of Epidemiology and Community Health 64, 213–215. 10.1136/jech.2009.090621 20203122

[55] Phillimore J., 2011 Approaches to health provision in the age of super-diversity: Accessing the NHS in Britain’s most diverse city. Critical Social Policy 31, 5–29.

[56] Brown P., & Gale N. (2018). Theorising risk work: Analysing professionals’ lifeworlds and practices. Professions and Professionalism, 8(1), e1988–e1988.

[57] Gale N., Dowswell G., Greenfield S., & Marshall T. (2017). Street-level diplomacy? Communicative and adaptive work at the front line of implementing public health policies in primary care. Social Science & Medicine, 177, 9–18 28152422 10.1016/j.socscimed.2017.01.046

[58] Charmaz K., 2014 Constructing grounded theory (2nd Edition). Sage, London.

[59] Timmermans S., Tavory I., 2012 Theory Construction in Qualitative Research: From Grounded Theory to Abductive Analysis. Sociological Theory 30, 167–186.

[60] Gale N., & Sultan H. (2013). Telehealth as ‘peace of mind’: Embodiment, emotions and the home as the primary health space for people with chronic obstructive pulmonary disorder. Health & place, 21, 140–147. 23474353 10.1016/j.healthplace.2013.01.006

[61] Hergenrather K.C., Rhodes S.D., Cowan C.A., Bardhoshi G., Pula S., 2009 Photovoice as a community-based participatory research: a qualitative review. Am J Health Behav 33. 10.5993/ajhb.33.6.6 19320617

[62] South East Public Health Observatory, 2012 Cardiovascular Disease Network Health Profile: Birmingham, Sandwell and Solihull. Public Health England.

[63] Vertovec S., 2007 Super-diversity and its implications. Ethnic and racial studies 30, 1024–1054.

[64] Phillips D., Harrison M., 2010 Constructing an integrated society: Historical lessons for tackling black and minority ethnic housing segregation in Britain. Housing Studies 25, 221–235.

[65] Phillips D., 2014 Segregation, mixing and encounter, in: Vertovec S. (Ed.), Routledge International Handbook of Diversity Studies, pp. 337–344.

[66] Kyle R., Blair A., 2007 Planning for health: generation, regeneration and food in Sandwell. International Journal of Retail & Distribution Management 35, 457–473.

[67] Martin K.N., Gallagher V.J., 2013 You make it amazing: the rhetoric of art and urban regeneration in the case of The Public. Journal of Visual Literacy 32, 51–73.

[68] Paynter F., 2010 Suburbs, culture and regeneration: cultural strategies in three English suburban boroughs. Queen Mary, University of London, London.

[69] Rex D., Blair A., 2003 Unjust des(s)erts: food retailing and neighbourhood health in Sandwell. International Journal of Retail & Distribution Management 31, 459–465.

[70] Viljoen A., 2005 Sandwell: a rich country and food for the poor, CPULs: Continuous Productive Urban Landscapes. Architectural Press Oxford, pp. 48–51.

[71] Marshall T., Westerby P., Chen J., Fairfield M., Harding J., Westerby R., Ahmad R., Middleton J., 2008 The Sandwell Project: A controlled evaluation of a programmes of targeted screening for prevention of cardiovascular disease in primary care. BMC Public Health 8, 73 10.1186/1471-2458-8-73 18298863 PMC2278139

[72] Papaloukas P., Quincey K., Williamson I.R., 2017 Venturing into the visual voice: combining photos and interviews in phenomenological inquiry around marginalisation and chronic illness. Qualitative research in psychology 14, 415–441.

[73] Blaxter M., 1990 Health and lifestyles. Routledge, London.

[74] Tavory I., Timmermans S., 2014 Abductive analysis: Theorizing qualitative research. University of Chicago Press.

[75] Blaxter M., 1997 Whose fault is it? People's own conceptions of the reasons for health inequalities. Social Science & Medicine 44, 747–756. 9080559 10.1016/s0277-9536(96)00192-x

[76] Popay J., Bennett S., Thomas C., Williams G., Gatrell A., Bostock L., 2003 Beyond ‘beer, fags, egg and chips’? Exploring lay understandings of social inequalities in health. Sociology of health & illness 25, 1–23. 14498942 10.1111/1467-9566.t01-1-00322

[77] van Loon J., 2014 Remediating risk as matter–energy–information flows of avian influenza and BSE. Health, risk & society 16, 444–458.

[78] Lipsky M., 1980 Street-Level Bureaucracy: Dilemmas of the Individual in Public Service. Russell Sage Foundation, New York.

[79] Mol A., Moser I., Pols J., 2015 Care in practice: On tinkering in clinics, homes and farms. transcript Verlag.

[80] Aranda K., Zeeman L., Scholes J., Morales A.S.-M., 2012 The resilient subject: Exploring subjectivity, identity and the body in narratives of resilience. Health: 16, 548–563. 10.1177/1363459312438564 22547553

[81] Mathers J., Taylor R., Parry J., 2016 Measuring the impact of Health Trainers Services on health and health inequalities: does the service's data collection and reporting system provide reliable information? Journal of Public Health. 10.1093/pubmed/fdv214 26819147

[82] South J., Woodward J., Lowcock D., 2007 New beginnings: stakeholder perspectives on the role of health trainers. The Journal of the Royal Society for the Promotion of Health 127, 224–230. 17970355 10.1177/1466424007081791

