# Supplementary material for: Risk work or resilience work? A qualitative study with community health workers negotiating the tensions between biomedical and community-based forms of health promotion in the United Kingdom
Source: PLoS One. 2019 Jul 29;14(7):e0220109. doi: 10.1371/journal.pone.0220109 (PMC6662997; doi:10.1371/journal.pone.0220109)
Supplement: S1 File — (DOCX) [file pone.0220109.s001.docx]

**Highlights**

- Public health interventions are increasingly delivered by community health workers (CHWs)
- CHWs are critical insiders, acknowledging the social determinants of health
- Within this context, they aim to achieve small but sustainable lifestyle changes
- They act as role models embodying healthy lifestyles
- The individualistic focus of the concepts of risk and resilience is not challenged
